# Plexiform tumorlet in a woman with postmenopausal endometrioid endometrial carcinoma taking tamoxifen for breast cancer: A case report

**DOI:** 10.1016/j.crwh.2022.e00459

**Published:** 2022-11-02

**Authors:** Nektarios Koufopoulos, Ioannis Boutas, Dionysios Dimas, Adamantia Kontogeorgi, Constantine Dimitrakakis

**Affiliations:** a Second Department of Pathology, Medical School, National and Kapodistrian University of Athens, Attikon University Hospital, 15772 Athens, Greece; b Breast Unit, Rea Maternity Hospital, P. Faliro, 17564 Athens, Greece; c Breast Unit, Athens Medical Center, Psychiko Clinic, 11525 Athens, Greece; d Third Department of Obstetrics and Gynecology, Medical School, National and Kapodistrian University of Athens, Attikon University Hospital, 15772 Athens, Greece; e First Department of Obstetrics and Gynecology, Medical School, National and Kapodistrian University of Athens, Alexandra University Hospital, 11528 Athens, Greece

**Keywords:** Epithelioid leiomyoma, Plexiform leiomyoma, Plexiform tumorlet, Uterus, Endometrioid carcinoma, Invasive lobular carcinoma

## Abstract

Leiomyomas are the most common benign smooth muscle tumors of the female genital tract. Among the recognized histologic variants, one of the least common is plexiform leiomyoma. When they are small, even microscopic findings, they are referred to as plexiform tumorlets.

In this report the authors describe a case of plexiform tumorlet found incidentally in the hysterectomy specimen of a 64-year-old woman with concomitant endometrioid endometrial carcinoma and a previous history of invasive lobular breast carcinoma. The endometrioid endometrial carcinoma arising on a polyps was diagnosed in a curettage specimen. In the hysterectomy specimen there was no residual endometrioid endometrial carcinoma. Six uterine leiomyomas were found. A small (3 mm) well circumscribed tumor was located in the uterine wall. Histologically it consisted of small nests, islands, cords, and tubules of relatively uniform, round to oval cells surrounded by loose stroma. The differential diagnosis was challenging, and included plexiform leiomyoma, uterine tumor resembling ovarian sex cord tumor, endometrial stromal tumor, and poorly differentiated endometrial or metastatic invasive lobular breast carcinoma. After careful morphologic evaluation and appropriate use of immunohistochemical stains the diagnosis of plexiform tumorlet was made.

## Highlights

• Plexiform leiomyoma is a rare subtype of epithelioid leiomyoma.
• Small size (under 1 cm) plexiform leiomyomas are referred to as plexiform tumorlets.
• Plexiform leiomyomas and tumorlets are diagnostically challenging due to their morphology and rarity.
• Plexiform leiomyomas and tumorlets may resemble histologically different uterine tumors

## 1 Introduction

Uterine leiomyomas are benign smooth muscle tumors found in 75% of hysterectomy specimens [1]. There are several histologic variants, including epithelioid leiomyoma, which can be further divided into leiomyoblastoma, clear cell leiomyoma, and plexiform leiomyoma. When their size is less than 1 cm or they are incidental microscopic findings, they are referred to as plexiform tumorlets.

Endometrioid endometrial carcinoma is the most common type of neoplasm of the uterine corpus, accounting for around 80% of all endometrial carcinomas [2].

Invasive lobular breast carcinoma is the most common special subtype of breast carcinoma, accounting for 5–15% of invasive breast carcinomas [3]. It tends to metastasize more frequently to the peritoneum, the gastrointestinal and female genital tract [4]. Metastatic involvement of the uterine corpus is rare [5]. It usually occurs in the myometrium and less commonly in the endometrium and leiomyomas [6,7]. Breast carcinoma is the most common metastatic neoplasm, with invasive lobular breast carcinoma accounting for 90% of cases [5].

We report here a diagnostically challenging case of plexiform tumorlet with concomitant endometrioid carcinoma arising on a polyp in a woman with a history of invasive lobular breast carcinoma and review the literature, focusing on the differential diagnostic issues posed by this rare entity. This is the second case of a plexiform leiomyoma or tumorlet in a patient with a previous history of invasive lobular breast carcinoma [5].

## 2 Case Presentation

**Fig. 1:**
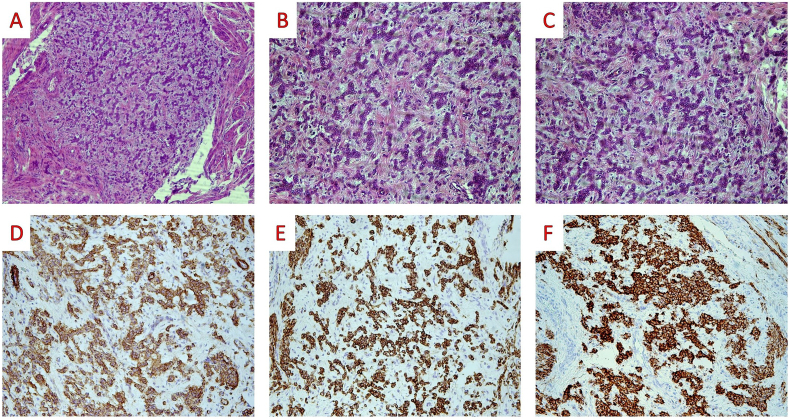
On medium-power examination, the tumor is nodular and well circumscribed (A: H&E X 100). On high-power examination, the tumor is seen to consist of small nests, islands, cords, and tubules of relatively uniform, round to oval cells surrounded by loose stroma (B, C: H&E X 200). Tumor cells stained for SMA, Desmin, and CD56 (D: SMA X 200; E: Desmin X 200; F: CD56 X 200).

A 64-year-old woman with a history of invasive lobular breast carcinoma grade 2 (estrogen and progesterone receptor-positive, Her-2 negative) stage pT2 N0(sn), three years previously, and presently taking tamoxifen, was admitted to the gynecology department with recurrent vaginal bleeding. Endometrial curettage was performed. The histological diagnosis was endometrioid carcinoma grade I arising in a polyp. A hysterectomy and bilateral salpingo-oophorectomy were performed. On microscopic examination, no residual endometrioid carcinoma was found. Six uterine leiomyomas with a maximum diameter ranging from 1.2 cm to 4.3 cm were found. A small, well-circumscribed tumor measuring 3 mm was also found in the uterine wall. It consisted of small nests, islands, cords, and tubules of relatively uniform, round to oval cells surrounded by loose stroma (Fig. 1). Mitotic figures, pleomorphism, and necrosis were absent. The differential diagnosis included plexiform tumorlet, uterine tumor resembling ovarian sex cord tumor (UTROSCT), and metastatic invasive lobular breast carcinoma.

Immunohistochemical study was positive for SMA, Desmin, CD56, and negative for AE-1/AE-3, EMA, Inhibin, Melan-A, WT-1, CD10, and CD-99.

The diagnosis was a plexiform tumorlet of the uterus based on morphology and immunohistochemical findings. Recovery was uneventful, and the woman was discharged on postoperative day 11. The multidisciplinary tumor board decided to administer anastrozole instead of tamoxifen for invasive breast carcinoma and not to administer adjuvant treatment for endometrial carcinoma. The woman was alive without evidence of recurrence or metastasis 42 months after surgery.

## 3 Discussion

Plexiform leiomyoma is an extremely rare variant of epithelioid leiomyoma [8]. It can occur in uterine and extrauterine locations [9,10]. Uterine plexiform leiomyoma is usually found in hysterectomy specimens taken for a variety of reasons, usually of a benign nature [8]. When these lesions are less than 1 cm in greatest diameter, they are referred to as plexiform tumorlets of the uterus [8]. Plexiform morphology can occur in the setting of disseminated peritoneal leiomyomatosis, intravenous leiomyomatosis, and diffuse uterine leiomyomatosis [8]. Only one case of plexiform epithelioid leiomyosarcoma has been reported [11].

Clinically, plexiform tumorlets are incidental findings due to their small size. They are multiple in around 25% of cases [8].

Regarding histogenesis, several speculations have been proposed. Based on immunohistochemical and ultrastructural findings, most authors consider plexiform leiomyomas to be of smooth muscle origin [12]. Other hypotheses suggest that they are of endometrial stromal or endothelial origin [13] or UTROSCT with myoid differentiation [12].

Histologically, they are nodular with well circumscribed or infiltrative borders [12]. They are composed of round or polygonal cells arranged in cords or clusters [8]. They express markers of smooth muscle differentiation such as SMA and desmin and CD56 on immunohistochemistry [5,8]. They may occasionally express epithelial markers.

The differential diagnosis includes UTROSCT, endometrial stromal tumor, and poorly differentiated endometrial or metastatic carcinoma.

UTROSCT has a variable histologic pattern, including small nests, anastomosing cords, broad trabeculae, glomeruloid or cystic structures, sertoliform or retiform tubules, and prominent Leydig-like cells [14]. The immunohistochemical profile is also diverse, expressing markers of sex cord differentiation such as calretinin, inhibin, CD99, and Melan-A and smooth muscle markers such as SMA, desmin, and caldesmon. Also, they may occasionally express epithelial markers or CD10 [8,14,15].

Endometrial stromal tumors may display sex cord and smooth muscle differentiation. In such cases, smooth muscle metaplasia shows epithelioid cells arranged in irregular islands or around a central hyalinized area, the so-called starburst pattern [8]. Immunohistochemically, they express CD10 and markers of sex cord and smooth muscle differentiation when harboring such metaplastic elements.

Poorly differentiated endometrial and metastatic carcinomas may show a variety of architectural patterns, including, among others, solid, trabecular, small nests, cords, and tubular. Tumor cells are epithelioid, showing at least moderate and usually high-grade atypia, several mitoses, and sometimes necrosis. Plexiform tumorlets may occasionally show only focal low-grade atypia and no necrosis. Immunohistochemically, carcinomas always express markers of epithelial differentiation at least focally, and the immunohistochemical expression of sex cord and smooth muscle markers is always negative [8].

In the present case, the main differential diagnostic concern was metastasis due to the previous history of breast carcinoma. In addition, on microscopic examination, the organoid pattern of the plexiform tumorlet closely mimicked the single file pattern of invasive lobular breast carcinoma. Furthermore, the loose stroma around epithelioid cells simulated desmoplastic reaction, and cytologic atypia was focally present. These morphological findings, coupled with the previous history of invasive lobular breast carcinoma, raised the possibility of metastasis. However, the well-circumscribed tumor borders, lack of pleomorphism or necrosis, and absence of mitotic figures argued against this diagnosis. The immunohistochemical study was particularly helpful in this case. Epithelial markers CKAE1/AE3 and EMA, as well as immunohistochemical markers consistent with UTROSCT and endometrial stromal sarcoma, were negative. In contrast, tumor cells were positive for markers of smooth muscle differentiation. CD56 positive staining has been reported in the literature [12].

Regarding prognosis, plexiform leiomyomas show benign clinical behavior and the prognosis is excellent. Thus, surgical treatment is adequate, and no adjuvant therapy is needed.

## 4 Conclusions

In summary, we have reported a diagnostically challenging case of plexiform tumorlet in a woman with a history of invasive lobular breast carcinoma, have reviewed the literature, focusing on differential diagnostic issues, and have discussed this rare entity's histogenesis. Plexiform leiomyomas and tumorlets are rare tumors with a unique, interesting, and diagnostically challenging morphology.
